# Pathogenic Microôrganisms, Including Bacteria and Protozoa

**Published:** 1911-01

**Authors:** 


**Pathogenic Microörganisms, including Bacteria and Protozoa.** A practical manual for students, physicians and health officers. By **William H. Park,** M.D., Professor of Bacteriology and Hygiene in the University and Bellevue Hospital Medical College, and Director of the Research Laboratory, Department of Health, New York City; and **Anna W. Williams,** M.D., Assistant Director of the Research Laboratory. Fourth edition. Octavo, 670 pages, with 196 illustrations and 8 full-page plates. Lea & Febiger, Publishers, Philadelphia and New York. 1910. (Cloth, $3.75, net.)

Any of our readers looking for a textbook which will be comprehensive, reliable, and representing the latest thought in this most important department of medicine, will be pleased with the work of Park and Williams. The enemies of medicine, and we should not forget that our enemies are both numerous and active, are persistent in their assertions that we know nothing about the cause of disease. On the other hand, all students of medicine know that we have much to learn upon this subject; but we may well be proud of this most practical and at the same time the most scientific phase of medicine.

We have read this book, modestly called a manual, with the definite impression that the authors bring to this work three valuable modes of preparation for the making of a good book. We name them in the order of importance: years of experience in teaching the subject; abundant opportunity to observe the sick in dispensary and hospital; occasion in a well conducted laboratory to study the life history of pathogenic organisms, to see, to test, to prove their several disease-causing power.

The work shows that the writers improved their opportunities for gaining knowledge and also have the gift of making the printed pages speak for them. To us the more attractive chapters are: "The Products of Bacterial Growth," "The Relation of Bacteria to Disease," and "The Bacteriology of Milk and its Relation to Disease."

We especially commend the book's treatment of the vaccine therapy of the immunisators, our old acquaintance and sometimes rival homeopathic treatment in its modern and ultrascientific dress of opsonins, anaphylactins, all made in the laboratory, much as the lady might say of her new hat,—made in Paris. Upon this prominent topic the writers speak with wisdom free from the taint of enthusiasm, hysteria, or commercialism. We heartily commend the work. H. R. H.

